# Clutter and Range Ambiguity Suppression Using Diverse Pulse Train in Pulse Doppler System

**DOI:** 10.3390/s18072326

**Published:** 2018-07-18

**Authors:** Jiacen Xu, Lixiang Ren, Huayu Fan, Erke Mao, Quanhua Liu

**Affiliations:** 1 School of Information and Electronics, Beijing Institute of Technology, Beijing 100081, China; jiacenxu@126.com (J.X.); maoerke@bit.edu.cn (E.M.); liuquanhua@bit.edu.cn (Q.L.); 2 Key Laboratory of Electronic and Information Technology in Satellite Navigation, (Beijing Institute of Technology), Ministry of Education, Beijing 100081, China; 3 Department of Electronic Engineering, Tsinghua University, Beijing 100084, China; fan_huayu@sina.com

**Keywords:** PD process, optimal filter, clutter suppression, ambiguity suppression, inverse filter

## Abstract

Pulse Doppler (PD) systems are widely used for moving target detection, especially in scenarios with clutter. Range ambiguity, which arises from fixed parameters in waveforms, is an inherent drawback in conventional systems. By using a diverse pulse train such as a train of coherent diverse phase coded pulses, these ambiguous peaks can be suppressed effectively but at the cost of sidelobe dispersions. In this work, a novel efficient PD process is proposed to suppress range ambiguity and detect moving targets under strong clutter. Poly-phase coded pulses are employed along with optimal receiving filters, by which the dispersed sidelobes are mitigated to a great extent. Moreover, a novel clutter suppression procedure is included in the PD process, by which strong clutter can be greatly suppressed. Well-designed receiving and inverse filters are employed. Simulation examples are presented to verify the theories. Compared with conventional methods, much better detection results are obtained for both near and remote targets, especially in scenarios with strong clutter.

## 1. Introduction

The pulse Doppler (PD) process is an important technique for detecting moving targets in scenarios with clutter. In a conventional PD system, a single coherent processing interval (CPI) consists of a series of identical pulses repeatedly transmitted at a fixed pulse repetition interval (PRI). By fast-time pulse compression [1,2] and slow-time Fast Fourier Transform (FFT) [1,2], the two-dimensional (2-D) range and Doppler resolution of targets can be obtained with low numerical complexity [3,4]. An inherent drawback for conventional systems is range ambiguity. Caused by the fixed parameters such as the PRI and intra-pulse modulation, the detection results of remote targets are ambiguous with units of PRI [1].

A traditional approach to solve this problem is by using multiple bursts of pulses with different PRIs. The real delay of an ambiguous target can be unraveled by the Chinese remainder theorem [1,2]. However, this approach requires a long processing time. Signal-to-noise ratio (SNR) losses are also caused since the pulses of different bursts cannot be accumulated coherently. Furthermore, ghosts or false targets may be generated in cases of multiple targets [5].

Another solution to this ambiguity problem is to use a diverse pulse train, where different pulses are modulated by different intra-pulse modulations [3,6]. Echoes are received by a series of receiving filters each allocated to a different pulse. Echo pulses can only pass through the corresponding filters. In this manner, the ambiguous range peaks can be suppressed efficiently. Moreover, the diverse pulse train contains inherent advantages in electronic countermeasures (ECM) environments. The agilities of transmitted pulses will significantly increase the difficulties of radar identification and recognition in the Electronic Intelligence (ELINT) systems [7,8].

The most widely used modulation type is phase coding. Compared with other modulations, phase-coded pulses are more agile and wide varieties of codes with favorable features can be chosen [9,10,11]. In a diverse phase-coded pulse train, different pulses exhibit the same mainlobe and various distributed sidelobes after fast-time pulse compression. Thus, after the slow-time FFT, the power of the mainlobes can be accumulated coherently while the power of the sidelobes is dispersed among the range-Doppler plane [12,13]. In normal cases, power of the dispersed sidelobes are relatively low and moving targets can be detected efficiently without range ambiguities. However, in scenarios with strong clutter, the detection of weak targets will be affected by the dispersed sidelobes of the strong clutter, especially when matched filtering is employed.

For the problem of strong clutter, traditional spatial processes can be used if an active phased array system is employed [1,2]. The clutter can be estimated from an individual range cell and cancelled via maximum signal-to-interference plus noise ratio solution [14]. For other traditional systems, time-domain processes can be employed. For example, targets can be estimated optimally based on the clutter model which is initially estimated by a constant phase pulse train [5]. By using well-designed receiving filters, similar sidelobe structures can be obtained by different pulses, which will mitigate the sidelobe dispersions to a great extent [12,13]. All these procedures only concern the case when the clutter matches with the receiving filter. However, in detecting remote targets, the strong near-range clutter will not match with the current receiving filter. Various structured clutter outputs will be obtained by different filters and the clutter power will be dispersed after slow-time FFT. Aiming at this problem, a joint range and Doppler optimization method is proposed at the expense of significantly increased computation [3].

In this work, we focus on range ambiguity suppression and moving target detection under strong clutter with medium to low pulse repetition frequency (PRF) PD systems. A novel PD process is proposed with low numerical complexity. Poly-phase coded pulses are employed along with optimal receiving filters, by which the sidelobe dispersions can be greatly mitigated. Moreover, a novel time-domain clutter suppression procedure is included in the process, by which the clutter dispersions in remote target detection can be eliminated efficiently. Well-designed receiving and inverse filters are employed. Compared with traditional methods, the novel PD process obtains much better detection results for both near and remote targets, especially in strongly cluttered conditions.

The organization of this work is as follows. Signal models and optimal filter design procedures are introduced at first in Section 2. Then, a basic PD process is presented. In Section 3, a clutter suppression procedure is presented. Based on the signal models, a receiving filter construction procedure is proposed. Then, a complete PD process is presented by combining the basic PD process with clutter suppression. In Section 4, simulation results are presented to verify the proposed method and conclusions are presented in Section 5.

## 2. Signal Model and Range Ambiguity Suppression

In this section, signal model and optimal filtering procedures are presented by which the dispersed sidelobes are mitigated greatly. Then, a basic PD process is presented, by which the range ambiguities can be suppressed efficiently.

### 2.1. Signal Model

Generally, the received echoes are processed in units of PRI for computation efficiency. Given a diverse pulse train with *K* different pulses, each pulse $s_{k}\left(n\right),\;\left(k=1\cdots K\right)$ is modulated by a different phase code sequence with *M* chips, $\left\{c_{0,k},c_{1,k},c_{2,k},\cdots ,c_{M-1,k}\right\}$. In each PRI, the transmitted pulse can be expressed as

(1) $$s_{k}\left(n\right)=\frac{1}{\sqrt{M}}\sum_{m=0}^{M-1}c_{m,k}u_{c}\left(n-mN_{c}\right)$$

where $N_{c}$ denotes the chip width and $u_{c}\left(n\right)$ denotes the envelope of each chip. Generally, for the maximization of transmitting power, a rectangular chip envelope is employed as follows:

(2) $$u_{c}\left(n\right)=\frac{1}{\sqrt{N_{c}}}\cdot \left\{ \begin{array}{l} 1,\;0\leq n<N_{c}\\ 0,\;\mathrm{otherwise} \end{array}\right.$$

Thus, the pulse spectrum is

(3) $$S_{k}\left(\omega \right)=\frac{1}{\sqrt{MN_{c}}}\sum_{m=0}^{M-1}c_{m,k}\exp\left(-j\omega mN_{c}\right)\frac{\exp\left(j\omega \left(N_{c}-1\right)/2\right)\sin\left(\omega N_{c}/2\right)}{\sin\left(\omega /2\right)}$$

Then, $S_{k}\left(\omega \right)$ can be written into two terms:

(4) $$S_{k}\left(\omega \right)=A_{k}\left(\omega \right)U_{k}\left(\omega \right)$$

where

(5) $$U_{k}\left(\omega \right)=\frac{\exp\left(j\omega \left(N_{c}-1\right)/2\right)\sin\left(\omega N_{c}/2\right)}{\sqrt{N_{c}}\sin\left(\omega /2\right)}$$

(6) $$A_{k}\left(\omega \right)=\frac{1}{\sqrt{M}}\cdot \sum_{m=0}^{M-1}c_{m,k}\exp\left(-jN_{c}\omega m\right)$$

Here, $U_{k}\left(\omega \right)$ is an envelope form of $S_{k}\left(\omega \right)$ that determines the total pulse bandwidth. Note that $U_{k}\left(\omega \right)$ has a smooth form and depends only on the chip width $N_{c}$. $A_{k}\left(\omega \right)$ is a ripple term caused by phase switching. Furthermore, (6) can be interpreted as the Fourier transform of a discrete series, where the sequence $\left\{c_{m,k}\right\}$ is obtained by sampling the *k*-th pulse at the rate of $N_{c}$.

Therefore, for simplicity, we can use the corresponding code sequence to represent the phase coded pulse. The real pulse can be simply obtained by convoluting the sequence with the chip envelope.

### 2.2. Optimal Receiving Filters

In general, the sidelobe levels of phased coded pulses are relatively high in their matched filter outputs, which will result in severe sidelobe dispersions after slow-time FFT, especially in scenarios with strong clutter. In order the mitigated the dispersion of sidelobes, optimal filters are employed at first in the fast-time pulse compression procedure.

As discussed in [15], sidelobes are caused by the ripples in the spectrum. In fact, there would be no sidelobes if the ripple term $A_{k}\left(\omega \right)$ was precisely compensated for. In that case, sidelobe dispersions would be eliminated completely. However, precise compensation is hard to obtain. The Z-transform of the code sequence is given as

(7) $$A_{k}\left(z\right)=\sum_{m=0}^{M-1}c_{m,k}z^{-n}$$

Thus, the ideal sidelobe suppression filter can be expressed as

(8) $$B_{k}\left(z\right)=\frac{1}{\sum_{m=0}^{M-1}c_{m,k}z^{-n}}$$

Unfortunately, (8) is unstable for most codes. A most common trade-off procedure is to approach this ideal filter by constructing a sequence with limited time duration. This filter is typically referred to as an optimal or mismatched filter. Compared with binary phase codes, poly-phase codes show a more gradual phase switching, making it much easier to compensate for the spectra ripples. In other words, a much lower output sidelobe level can be obtained by poly-phase codes [16].

Various optimal filter design procedures can be applied to poly-phase codes. For example, a minimum integrated sidelobe level (ISL) filter can be obtained in closed form by solving a least square (LS) problem [17,18] or Cauchy-Schwartz inequality [19]. By iterating the ISL results [18] or other optimization procedures [19,20], a minimum peak sidelobe level (PSL) filter can be obtained. In the following process, we employ optimal ISL filters as receiving filters since optimal PSL filters are used only in scenarios when discrete strong scattering exists. For scenarios dominated by distributed clutter, which are much more common, optimal ISL filters can achieve much better estimation and detection results [19].

Suppose that the optimal ISL filter consists of *P* chips. In vector form, the code sequence $s_{k}$ and the optimal filter sequence $h_{k}$ can be expressed as

(9) $$\begin{array}{l} s_{k}={\left[\begin{array}{cccccc} 0^{T} & c_{0,k} & c_{1,k} & \cdots & c_{M-1,k} & 0^{T} \end{array}\right]}^{T}\\ h_{k}={\left[\begin{array}{cccc} h_{0,k} & h_{1,k} & \cdots & h_{P-1,k} \end{array}\right]}^{T} \end{array}$$

where zeros are padded before and after the code sequence, **0** is an all-zero vector of size (P−M)/2×1 and the superscript ^T^ denotes the vector transpose. Then, the Hankel matrix $X_{k}$ of the code $s_{k}$ can be written as

(10) $$X_{k}=[\begin{array}{ccccccc} 0 & \cdots & 0 & s_{0,k} & s_{1,k} & \cdots & s_{P-1,k}\\ \vdots & \ddots & & \cdots & & & 0\\ 0 & s_{0,k} & s_{1,k} & \cdots & s_{P-1,k} & 0 & \vdots \\ s_{0,k} & s_{1,k} & \cdots & s_{P-1,k} & 0 & \cdots & 0 \end{array}{]}_{(2P-1)\times 1}$$

The output $y_{k}$ of the optimal filter can be expressed as

(11) $$y_{k}=X_{k}^{H}h_{k}$$

The ISL is defined as

(12) $${\mathrm{ISL}=10\log}_{10}\frac{\sum_{p\neq P}{\left|y_{k}\left(p\right)\right|}^{2}}{{\left|h_{k}^{H}s_{k}\right|}^{2}}$$

Thus, the optimal ISL filter can be obtained by solving the following optimization problem

(13) $$\begin{array}{c} \min{\left\|y_{k}-y_{N}\right\|}_{2}^{2}\\ \mathrm{subject}\;\mathrm{to}\;s_{k}^{H}h_{k}=M \end{array}$$

where $y_{M}$ is an ideal output

(14) $$y_{M}={\left[\begin{array}{ccc} 0_{d}^{T} & M & 0_{d}^{T} \end{array}\right]}^{T}$$

Here, $0_{d}$ is an all-zero vector of (P−1)×1 size.

Using the Lagrangian multiplier method, the solution to this optimization problem can be expressed in closed form as

(15) $$h_{k}=\frac{M{\left(X_{k}X_{k}^{H}\right)}^{-1}s_{k}}{s_{k}^{H}{\left(X_{k}X_{k}^{H}\right)}^{-1}s_{k}}$$

In this manner, the real optimal filter can be obtained by convoluting with the chip envelope

(16) $$h_{k}\left(n\right)=\frac{1}{\sqrt{P}}{\tilde{h}}_{k}\left(n\right)*u_{c}\left(n\right)$$

Here, ∗ denotes the convolution approach and ${\tilde{h}}_{k}\left(n\right)$ is a zero-interpolated form of the filter sequence $h_{k}$.

(17) $${\tilde{h}}_{k}\left(n\right)=\left\{ \begin{array}{cc} h_{n/N_{c},k} & ,\;\mathrm{if}\;n\;\mathrm{is}\;a\;\mathrm{multiple}\;\mathrm{of}\;N_{c}\\ 0 & ,\;\mathrm{if}\;n\;\mathrm{is}\;\mathrm{not}\;a\;\mathrm{multiple}\;\mathrm{of}\;N_{c} \end{array}\right.$$

Using optimal filters $h_{k}\left(n\right)$, very low sidelobe levels can be obtained after fast-time pulse compression.

### 2.3. Basic PD Process

In this section, a basic PD process with diverse pulse train is presented using optimal receiving filters. This basic process is a major component of the novel PD process which is presented in detail in Section 3.2.

In tradition PD process with identical pulses, the range-Doppler resolution of a target is initially obtained by fast-time pulse compression within each PRI, followed by a slow-time FFT among the PRIs [1]. The range coverage $R_{c}$ of the output range-Doppler plane is $cT_{r}/2$, where $T_{r}$ denotes the PRI.

Such procedures also apply to the case of diverse pulse train. Target echoes are pulse compressed into narrow peak forms by corresponding receiving filters in the fast-time domain. Then, same slow-time FFT can be applied. Furthermore, for the calculation efficiency, we divide the maximum radar range $R_{total}$ into *L* subintervals with units of $R_{c}$, where $R_{total}=L\cdot R_{c}$. In this manner, fast-time pulse compression only needs to be applied within each $T_{r}$. Different subintervals can be selected by simply adjusting the delay of receiving filter train. For example, targets contained by the *i*-th (*i* = 1,⋯*L*) subinterval can be detected by delaying the receiving filters train for $\left(i-1\right)\cdot T_{r}$. Echoes of targets in other subintervals cannot pass through the current receiving filters. By jointing the range-Doppler planes of all the subintervals, we can obtain the unambiguous 2-D resolution of range and Doppler within the radar range.

Now we will present the procedure of fast-time pulse compression and slow-time FFT in sequence form. The ideal received pulses and receiving filters are expressed as (9). Then the filter outputs can be expressed as

(18) $$y={\left[\begin{array}{cccc} y_{1} & y_{2} & \cdots & y_{K} \end{array}\right]}_{\left(2P-1\right)\times K}$$

According to the constraint condition in (15), we have

(19) $$y_{k}\left(P\right)=h_{k}^{H}s_{k}=M$$

Obviously, different codes will result in different sidelobe structures, while the main peaks remain the same.

Then, in the slow-time procedure, y is first weighted by a window function: W. Here, W is a K×K diagonal matrix with its diagonal elements being a window function, such as the Hamming and Blackman windows. The weighted form $y_{w}$ can be expressed as

(20) $$y_{w}={\left[yW,0_{w}\right]}_{\left(2P-1\right)\times Q}$$

Here, $y_{w}$ is padded to *Q* columns to obtain a zoomed spectrum in the following FFT procedure. $0_{w}$ is an all-zero matrix of size (2P−1)×(Q−K).

The slow-time FFT can be expressed as

(21) $$y_{PD}=F^{T}y_{w}$$

where F denotes the Fourier transform matrix

(22) $$F=\left[\begin{array}{ccccc} 1 & 1 & 1 & \cdots & 1\\ 1 & e^{-j2\pi /Q} & e^{-j2\pi 2/Q} & \cdots & e^{-j2\pi \left(Q-1\right)/Q}\\ \vdots & \vdots & \vdots & & \vdots \\ 1 & e^{-j2\pi \left(L-1\right)/Q} & e^{-j2\pi 2\left(Q-1\right)/Q} & \cdots & e^{-j2\pi \left(Q-1\right)\left(Q-1\right)/Q} \end{array}\right]$$

Thus, in the slow-time procedure, the power of the main peaks can be accumulated coherently, whereas the sidelobes are dispersed among the range-Doppler plane in a manner similar to random noise. Since the sidelobes have already been suppressed to very low levels, the levels of dispersed sidelobes are mitigated greatly in this basic PD process over that of conventional approach.

Note that discrete targets contained by other subintervals cannot be detected in current subinterval since their echoes cannot be pulse compressed by current receiving filter train. After slow-time FFT, their echo power will be dispersed among the range-Doppler plane. This dispersed power is very small and has little effect on target detection in current subinterval. In this manner, the range ambiguity can be efficiently suppressed.

**Figure 1:**
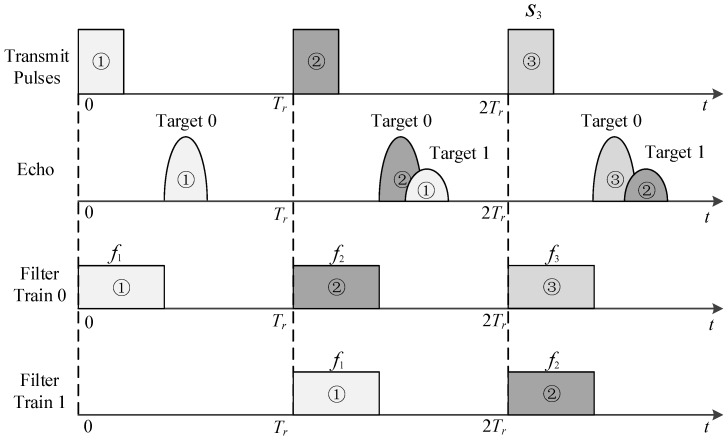
Echo and reference pulse diagram for a diverse pulse train.

In Figure 1, we present a diagram for the receiving process. Suppose that two stationary targets are contained in the scenario: a near strong Target 0 and a far weak Target 1. Obviously, range ambiguity will exist for Target 1 if identical pulses are used. In contrast, the two targets can be detected separately in a diverse pulse system. In the diagram, we mark the corresponding transmit pules, echoes and receiving filters with a same number. Here Filter train 0 is set to detect targets in the first subinterval, such as Target 0. Filters in each PRI only correspond with echoes of near range targets. Target echoes in other subintervals cannot be pulse-compressed, such as Target 1. When we delay this filter train by *T_r_*, as is shown by the Filter train 1 in the bottom, targets within the time range of *T_r_* to 2*T_r_* can be detected, such as Target 1. Similarly, Target 0 cannot be detected by filter train 1.

## 3. Clutter Suppression and PD Process

In this section, we present a clutter suppression procedure, by which the near-range strong clutter can be suppressed efficiently. This procedure is conducted in parallel with the basic PD procedure. Then, a complete PD process is proposed by combining the basic PD process and the clutter suppression procedure.

### 3.1. Clutter Suppression Using Diverse Pulse Train

In some scenarios, strong clutter may exist in the near range, especially in the first subinterval. Fortunately, in a cluttered subinterval, the majority of the clutter power is accumulated in several resolution bins with low velocity after the basic PD process. Moving targets can be separated from the clutter easily via their differences in the Doppler domain. Remote clutter and target echoes in other subintervals do not match with the current receiving filters and their power will be dispersed among the current range-Doppler plane. This dispersed power is relatively small and has little effect on moving target detection in current subinterval.

However, when we adjust the filter delays to choose other subintervals, the near-range clutter will also be dispersed since it does not match with the receiving filters. This dispersed clutter is relatively strong and will affect the target detection.

To address this problem, we propose a clutter suppression procedure in parallel with the basic PD procedure. The clutter suppression procedure consists of three steps. The first step is similar to the basic PD process. Received data are pulse compressed in fast-time domain by well-designed optimal receiving filters. These filters are slightly different from the basic PD case. Then same slow-time FFT are applied to the pulse compressed data using a slightly weighted window. Next, in the second step, the clutter is suppressed by zeroing the resolution bins dominated by the clutter. Target echoes from other subintervals will be dispersed among the current range-Doppler plane. Therefore, zeroing few resolution bins has slight influences on these dispersed target echoes. Finally, in the third step, the clutter suppressed data are restored to time domain. Slow-time Inverse Fast Fourier Transform (IFFT) are employed at first, followed by an inverse filtering process in fast-time domain using well-designed inverse filters.

After this suppression procedure, we can move on to the next subintervals by changing the delay of receiving filter train. Then the basic PD process can be applied again. In this manner, much better target detecting results can be obtained in the remaining subintervals.

Obviously, receiving filter design is a key factor in the clutter suppression. We need to maintain the output sidelobe levels as well as guarantee the stabilities of the corresponding inverse filters. However, the inverse filters will be unstable if we invert the original receiving filters $h_{k}\left(n\right)$ directly. Therefore, in the clutter suppression procedure, we employ another group of receiving filters, $g_{k}\left(n\right)$, whose output range resolution and sidelobe levels are very similar to $h_{k}\left(n\right)$. More importantly, the corresponding stable inverse filters can be obtained by inverting the receiving filters directly.

In Z-domain, an ideal inverse filter for $h_{k}\left(n\right)$ is obtained by the reciprocals of ${\tilde{h}}_{k}\left(n\right)$ and $u_{c}\left(n\right)$. Since ${\tilde{h}}_{k}\left(n\right)$ is an approximation of the inverse spectrum of the code sequence, no zero point exists on the unit circle of ${\tilde{h}}_{k}\left(n\right)$. However, as for the rectangle envelope $u_{c}\left(n\right)$, there are $N_{c}-1$ zero points on the unit circle, which would cause the inverse filter to be unstable if we were to invert $h_{k}\left(n\right)$ directly. Therefore, in order to avoid these zeros, we employ an exponent chip envelope to replace $u_{c}\left(n\right)$ in constructing $g_{k}\left(n\right)$. The chip envelope is expressed as

(23) $$u_{e}\left(n\right)=\left\{ \begin{array}{cc} e^{\frac{n}{N_{c}-1}}-1 & ,\;0\leq n<N_{c}-1\\ e^{\frac{2N_{c}-2-n}{N_{c}-1}}-1 & ,\;N_{c}-1\leq n<2N_{c}-2\\ 0 & ,\;\mathrm{otherwise} \end{array}\right.$$

The normalized exponent envelope is obtained by

(24) $$u_{ne}\left(n\right)=\frac{u_{e}\left(n\right)}{\sqrt{\sum_{n=0}^{2N_{c}-2}u_{e}^{2}\left(n\right)}}$$

Thus, similar to (16), the receiving filter $g_{k}\left(n\right)$ can be obtained by

(25) $$g_{k}\left(n\right)=\frac{1}{\sqrt{P}}{\tilde{h}}_{k}\left(n\right)*u_{ne}\left(n\right)$$

Then, the corresponding inverse filters can be obtained directly as

(26) $$v_{k}\left(n\right)=Z^{-1}\left(\frac{1}{Z\left(g_{k}\left(n\right)\right)}\right)$$

where Z(·) denotes the Z-transform and $Z^{-1}\left(\cdot \right)$ denotes the inverse Z-transform.

Although $g_{k}\left(n\right)$ is in different form with $h_{k}\left(n\right)$ in time domain, the outputs of the two filters are very similar. In fact, both of $h_{k}\left(n\right)$ and $g_{k}\left(n\right)$ contain a same ${\tilde{h}}_{k}\left(n\right)$. The spectrum ripples of the received phase coded pulse are compensated to the same degree. Therefore, the output trends of the two filters remain the same. Slight differences exist only in the envelope of each peak in the mainlobes and sidelobes.

**Figure 2:**
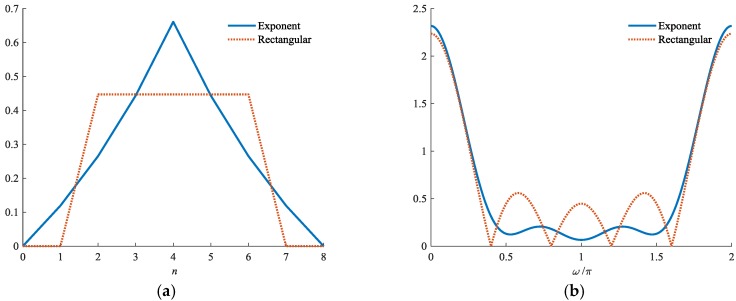
Chips with rectangular and exponent envelopes in: (**a**) time and (**b**) frequency domains, respectively.

**Figure 3:**
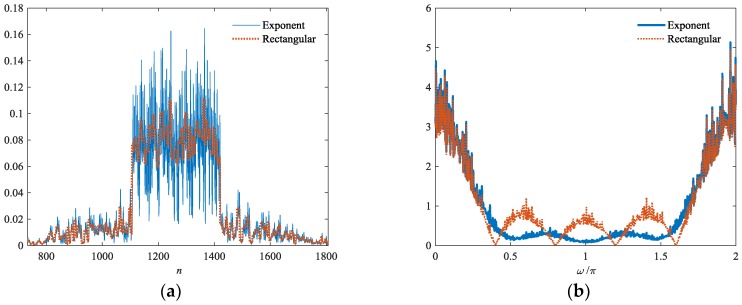
Envelopes of $h_{k}\left(n\right)$ and $g_{k}\left(n\right)$ in: (**a**) time and (**b**) frequency domains, respectively.

In Figure 2, we present examples of $u_{c}\left(n\right)$ and $u_{ne}\left(n\right)$ when $N_{c}=5$. For the ease of comparison, $u_{c}\left(n\right)$ is delayed by 2 sampled points in figure plotting. As shown in Figure 3b, zero points in the chip spectrum are efficiently avoided by the exponent envelope.

In Figure 3, the envelopes of $h_{k}\left(n\right)$ and $g_{k}\left(n\right)$ in the time and frequency domains are presented. The two filters are designed for the same poly-phase coded pulse with 63 chips. Both filters contain 504 chips. As shown in Figure 3b, zero points are successfully avoided in the filter spectrum. In this manner, the stability of the corresponding inverse filters is ensured.

**Figure 4:**
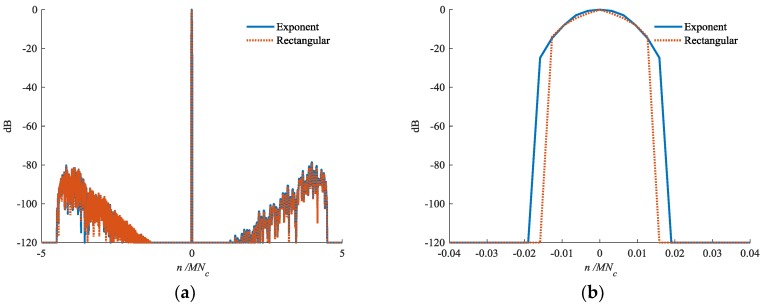
Output of $h_{k}\left(n\right)$ and $g_{k}\left(n\right)$ in: (**a**) Overall view and (**b**) Mainlobe area, respectively.

In Figure 4, we present the output results of $h_{k}\left(n\right)$ and $g_{k}\left(n\right)$. The chip envelope of the transmitted pulse is rectangular, as shown by (1). The output results of the two filters are very similar in the sidelobes. The mainlobe for $g_{k}\left(n\right)$ is broadened slightly, which is negligible compared with the stability of the corresponding inverse filter.

As for the slow-time procedure, unlike the basic PD case, no zero is padded in the slow-time domain. The corresponding slow-time inverse procedure is much easier. The FFT approach (21) can be inverted directly by the IFFT approach. The slow-time windowing can be inverted by dividing the window function directly. Since zeros are obtained at the start and end edges by highly weighted windows, in the clutter suppression procedure, slightly weighted windows are employed, such as Hamming windows.

### 3.2. PD Process Combined with Clutter Suppression

In this section, a complete PD process is presented by combining the basic PD process and the clutter suppression procedure.

The whole process is separated into two parts: a main PD loop and a parallel clutter suppression procedure. The output data of the clutter suppression procedure is saved for data updating in the next main loop. Then the clutter suppressed data can be used for the basic PD process.

**Figure 5:**
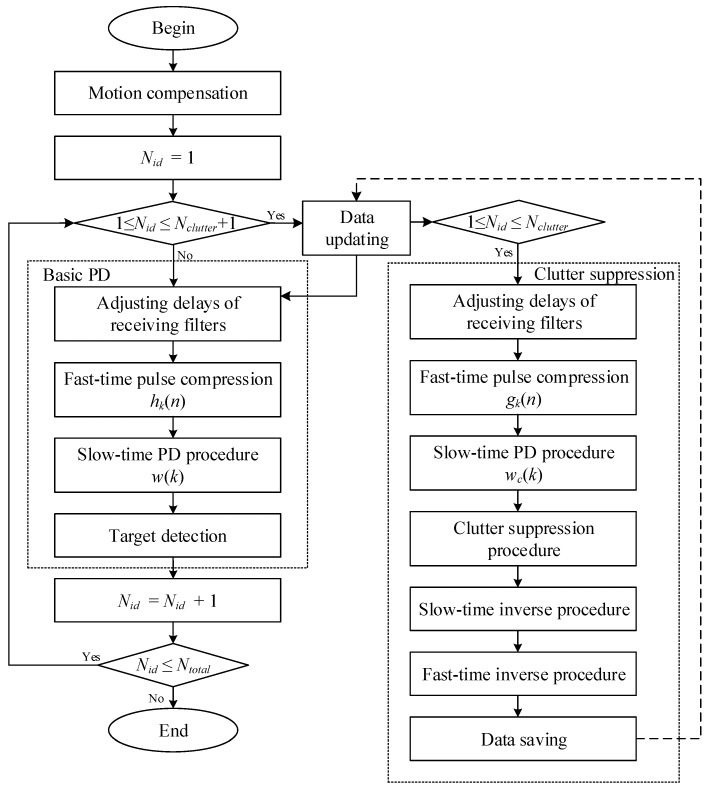
Pulse Doppler (PD) process flowchart.

The flowchart is shown in Figure 5. Let $N_{id}$ be the current subinterval, $N_{clutter}$ be the number of subintervals dominated by clutter and $N_{total}$ be the maximum subinterval number.

Detailed descriptions for the main PD process loop are as follows:

1. Initialize the scheme parameters and compensate the motion of the platform [1,2].
2. Choose the initial subinterval *N_id_* = 1.
3. Determine whether the condition of data loading 1 < *N_id_* ≤ *N_clutter_* + 1 is satisfied. Go to subsequent operations directly if the condition fails. Otherwise, update the data. Then, go to subsequent operations and apply the parallel clutter suppression procedure. If *N_id_* = 1, load the original received echoes. If 1 < *N_id_* ≤ *N_clutter_* + 1, update the data using the clutter-suppressed data from the clutter suppression procedure.
4. Adjust the delays of the receiving filters *h_k_*(*n*) to match the current subinterval *N_id_*.
5. Pulse compression in the fast-time domain.
6. Add the heavily weighted window *w*(*k*) in the slow-time domain and apply the FFT procedure.
7. Apply 2-D detections in the range-Doppler plane.
8. Move to the next subinterval, *N_id_* = *N_id_* + 1.
9. If *N_id_* ≤ *N_total_*, go to step 3. Otherwise, terminate the process.

Detailed descriptions for the clutter suppression procedure are as follows:

1. Determine whether the clutter suppression condition of 1 < *N_id_* ≤ *N_clutter_* is satisfied; if so, then go to the subsequent operations.
2. Adjust the delay of the receiving filters *g_k_*(*n*) to match the current subinterval *N_id_*.
3. Pulse compression in the fast-time domain.
4. Add slightly weighted windows *w_c_*(*k*) in slow-time domain and apply the FFT procedure.
5. Suppress the strong clutter by zeroing resolution bins dominated by the clutter.
6. Apply the inverse procedure in the slow-time domain.
7. Apply the inverse procedure in the fast-time domain.
8. Save the output data to be used in the next main PD process loop.

## 4. Simulation Results

In this section, simulation results are presented to verify the theories.

A diverse pulse train is employed as the transmit pulse train in the simulation. The train consists of 64 pulses with each modulated by a different poly-phase code with 63 chips. The chips in transmitted pulses are rectangular so as to maximize the transmit power. Pulses have a width of 25.2 μs and are transmitted at a PRI of 250 μs.

An airborne platform is employed. The flight height is 7 km and the velocity is 100 m/s. The antenna look-down angle is 20° and azimuth angle is 90° from ahead. Note that with this side-looking antenna, sidelobe clutters will cover the most resolution bins along the Doppler direction [1]. Better simulation results will be obtained if the antenna is rotated to another azimuth angle. Parameters for the transmitted pulse train and the platform are presented in Table 1.

The Morchin clutter model is used in the clutter simulation, where mountainous parameters are used in calculating the clutter Radar Cross Section (RCS) [1]. The distribution of clutters is Gaussian with a variance of 0.01. The coverage area of the clutters is two subintervals. Zero-mean white Gaussian noise is employed with its power 65 dB less than the maximum clutter power.

Nine moving targets are employed in the scenario. Specific target parameters of ranges, velocities and echo power relative to the maximum clutter power are presented in Table 2.

Three PD process simulation results are presented, including the traditional PD process, the basic PD process and the complete PD process. The result of signal-to-clutter and noise ratio (SCNRs) for each target is also presented in Table 2.

In the traditional PD process, matched filters are used as receiving filters in fast-time procedure and a Blackman window is used as w(k) in the slow-time procedure. The basic PD process is conducted as is described in Section 2.3. Optimal filters $h_{k}\left(n\right)$ with rectangular chip envelopes are employed in fast-time procedure and the same Blackman window is employed. No clutter suppression is included in this case. Based on the basic PD process, a complete PD process is conducted by employing the clutter suppression procedure. A Hamming window is used as $w_{c}\left(k\right)$ and an exponent envelope is used for $g_{k}\left(n\right)$ in the clutter suppression procedure.

**Figure 6:**
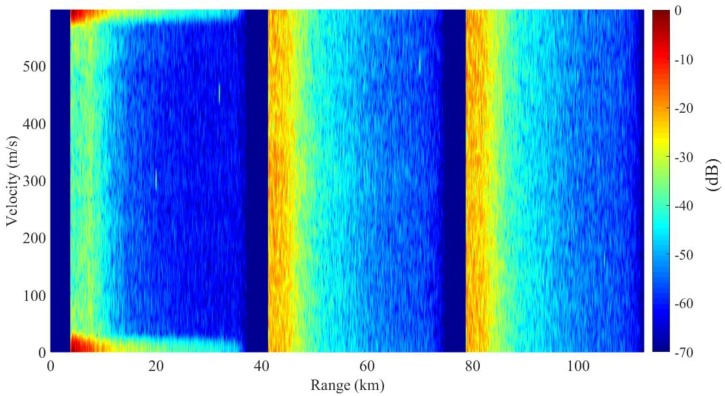
Range-Doppler resolution for traditional PD process using matched filters.

**Figure 7:**
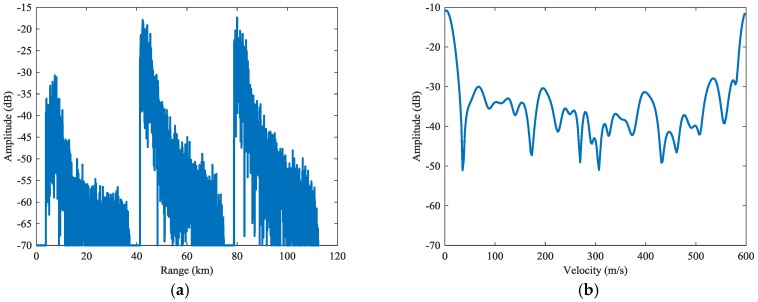
Cross profiles of Target 1 for traditional PD process: (**a**) range profile, (**b**) Doppler profile.

In Figure 6, we present the result of traditional PD process, where the sidelobes are dispersed severely in the first subinterval due to the high sidelobes of matched filter. In Figure 7, the range and Doppler profiles of Target are presented. This target is covered by the dispersed sidelobes completely. In the rest subintervals, the clutter is also severely dispersed and most of the target is lost. The SCNRs of the targets are very low, which are presented by SCNRs (1) in Table 2.

**Figure 8:**
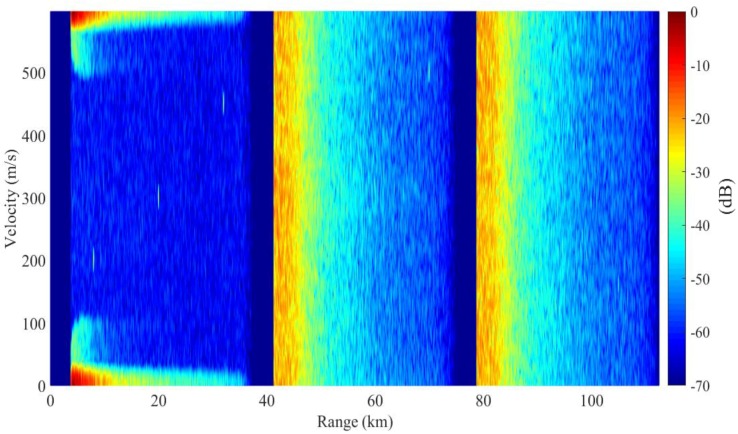
Range-Doppler resolution for the basic PD process.

**Figure 9:**
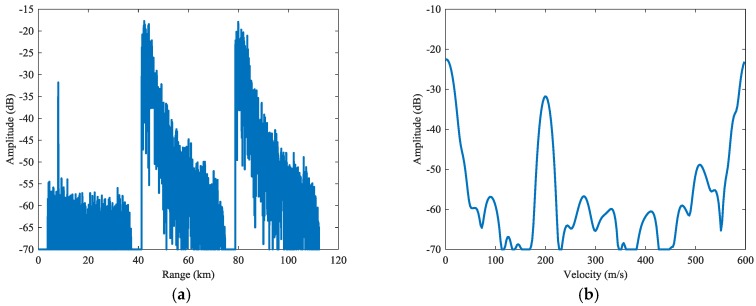
Cross profiles of Target 1 for the basic PD process: (**a**) range profile, (**b**) Doppler profile.

The result of basic PD process is presented in Figure 8. Due to the optimal filtering, sidelobe dispersions are mitigated greatly compared with the traditional PD. The SCNRs of the targets in the first subintervals has been improved by one to three times. As is shown in Figure 9, the target can be detected at an SCNR of 36.7 dB. However, the cutter dispersions are still severe in rest subintervals, as is shown by the SCNRs (2) in Table 2, the SCNRs of targets in these subintervals are still low.

**Figure 10:**
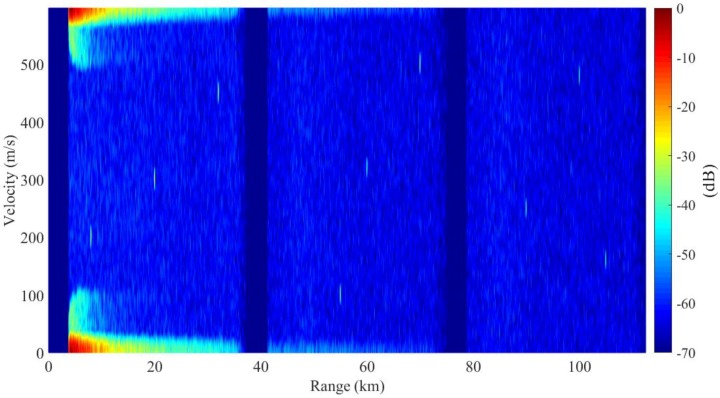
Range-Doppler resolution for the complete PD process.

**Figure 11:**
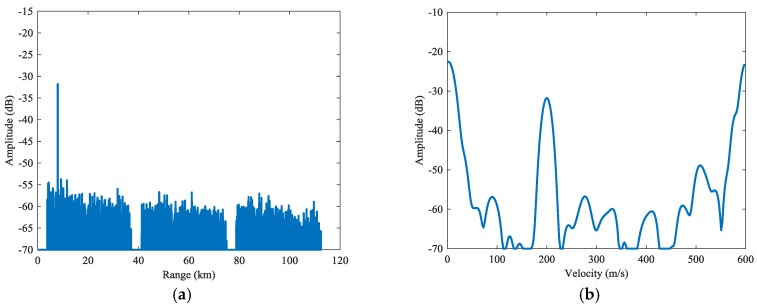
Cross profiles of Target 1 for the complete PD process: (**a**) range profile, (**b**) Doppler profile.

The result of complete PD process is presented in Figure 10. The range and Doppler profiles of the first target are presented in Figure 11. Compared with the basic PD case, the dispersed clutter is efficiently suppressed in remote intervals. In the detection region, the remaining clutter power is less than −65 dB. The SCNRs of the targets in the remote subintervals has been improved by one to three times. A specific SCNR result for each target is presented by SCNRs (3) in Table 2. Compared with the other two processes, the remote targets can be detected at much higher SCNRs.

## 5. Conclusions

In this work, we present a novel PD process using a diverse phase coded pulse train, by which the ambiguous peaks are efficiently suppressed. Sidelobe dispersions are mitigated to a great extent by employing poly-phase coded pulses and optimal ISL filters. A basic PD process is presented based on the diverse pulse train structure. A novel clutter suppression procedure is presented based on inverse filtering to obtain improved detection results in scenarios with strong clutter. A novel filter design procedure is included in this procedure, by which the stability of corresponding inverse filters is ensured. By combining the basic PD process with the clutter cancellation procedure, the complete PD process is presented. Verified by the simulations, the complete PD process can obtain much better detection results for both near and remote targets compared with the traditional PD process.

## Figures and Tables

**Table 1:** Parameters for the transmit signal and the platform.

Parameter	Value
Pulse width	25.2 μs
Chip width	0.4 μs
$N_{c}$	63
P	504
PRI	250 μs
K	64
*L*	256
Carrier frequency	1 GHz
Sample rate	10 MHz
Chip shape	Rectangular
Platform height	7 km
Platform velocity	100 m/s
Antenna aitch	20°
Antenna azimuth ^1^	90°

^1^ Azimuth angle form ahead.

**Table 2:** Target parameters and SCNRs.

No.	Range (km)	Velocity (ms^−^^1^)	Echo Power (dB)	SCNR (1) ^1^ (dB)	SCNR (2) ^2^ (dB)	SCNR (3) ^3^ (dB)
1	8	200	−32	10.42	31.77	31.77
2	20	300	−31	31.00	34.10	34.10
3	32	450	−35	29.78	30.30	30.30
4	55	100	−37	12.22	11.40	27.69
5	60	320	−38	14.48	13.83	27.22
6	70	500	−36	23.79	23.36	29.58
7	90	250	−40	8.54	8.67	25.10
8	100	480	−39	15.62	15.40	26.58
9	105	160	−41	16.87	17.12	25.20

^1^ SCNRs of the traditional PD process using matched filters. ^2^ SCNRs of the basic PD process. ^3^ SCNRs of the PD process with clutter suppression.

## References

[1] Schleher D.C. (2010). MTI and Pulsed Doppler Radar with MATLAB.

[2] Alabaster C. (2012). Pulse Doppler Radar: Principles, Technology, Applications.

[3] Scholnik D.P. Range-ambiguous clutter suppression with Pulse-diverse waveforms. Proceedings of the 2011 IEEE Radar Conference.

[4] Skolnik M.I. (2008). Radar Handbook.

[5] Lin F., Steiner M. New techniques for radar coherent range ambiguity resolution. Proceedings of the 2001 IEEE Radar Conference.

[6] Chung W., Wong K.T. Pulse-diverse radar waveform design for accurate joint estimation of time delay and Doppler shift. Proceedings of the ICASSP.

[7] Matuszewski J. Specific emitter identification. Proceedings of the IRS.

[8] Matuszewski J. The Radar Signature in Recognition System Database. Proceedings of the 19th International Conference on MIKON.

[9] Hu C., Li Y., Dong X., Wang R., Cui C. (2017). Optimal 3D deformation measuring in inclined geosynchronous orbit SAR differential interferometry. Sci. China (Inf. Sci.).

[10] Zeng T., Chang S., Fan H., Liu Q. (2018). Design and Processing of a Novel Chaos-Based Stepped Frequency Synthesized Wideband Radar Signal. Sensors.

[11] Zhou C., Liu F., Liu Q. (2017). An Adaptive Transmitting Scheme for Interrupted Sampling Repeater Jamming Suppression. Sensors.

[12] Blunt S.D., Cook M.R., Stiles J. Embedding information into radar emissions via waveform implementation. Proceedings of the 2010 International Waveform Diversity and Design Conference.

[13] Cook M.R., Blunt S.D., Jakabosky J. Optimization of waveform diversity and performance for pulse-agile radar. Proceedings of the Radar Conference.

[14] Higgins T., Gerlach K., Shackelford A.K., Blunt S.D. Aspects of Non-Identical Multiple Pulse Compression. Proceedings of the IEEE Radar Conference.

[15] Rihaczek A.W., Golden R.M. (1971). Range Sidelobe Suppression for Barker Codes. IEEE Trans. Aerosp. Electron. Syst..

[16] Sahin C., Metcalf J.G., Blunt S.D. Filter design to address range sidelobe modulation in transmit-encoded radar-embedded communications. Proceedings of the Radar Conference.

[17] Ackroyd M.H., Ghani F. (1973). Optimum mismatched filters for sidelobe suppression. IEEE Trans. Aerosp. Electron. Syst..

[18] Griep K.R., Ritcey J.A., Burlingame J.J. (1995). Poly-phase codes and optimal filters for multiple user ranging. IEEE Trans. Aerosp. Electron. Syst..

[19] Stoica P., Li J., Xue M. (2008). Transmit codes and receive filters for radar. IEEE Signal Process. Mag..

[20] Kajenski P.J. (2016). Mismatch filter design via convex optimization. IEEE Trans. Aerosp. Electron. Syst..

